# Measurement of protein digestibility in humans by a dual-tracer method

**DOI:** 10.1093/ajcn/nqy062

**Published:** 2018-05-15

**Authors:** Sarita Devi, Aneesia Varkey, M S Sheshshayee, Thomas Preston, Anura V Kurpad

**Affiliations:** 1 Division of Nutrition, St John's Research Institute, St John's National Academy of Health Sciences, Bangalore, India; 2 Department of Physiology, St John's Research Institute, St John's National Academy of Health Sciences, Bangalore, India; 3 Department of Crop Physiology, University of Agricultural Sciences, Bangalore, India; 4 Scottish Universities Environmental Research Centre, East Kilbride, United Kingdom

**Keywords:** protein digestibility, legumes, spirulina, stable isotopes

## Abstract

**Background:**

Recent evaluations of the risk of dietary protein deficiency have indicated that protein digestibility may be a key limiting factor in the provision of indispensable amino acids (IAAs), particularly for vulnerable populations living in challenging environments where intestinal dysfunction may exist. Since the digestion of protein occurs only in the small intestine, and the metabolic activity of colonic bacteria confounds measurements at the fecal level, there is a need to develop noninvasive protein digestibility measurements at the ileal level.

**Objective:**

We used a dual-tracer method with stable isotopes to characterize the digestibility of uniformly labeled [^13^C]-spirulina protein as a standard protein, in comparison to a mixture of ^2^H-labeled crystalline amino acids, and then demonstrated the use of this standard protein to measure the digestibility of selected legumes (chick pea and mung bean) through the use of proteins that were intrinsically labeled with ^2^H.

**Design:**

The digestibility of uniformly labeled [^13^C]-spirulina was first measured in 6 healthy volunteers (3 males and 3 females) by feeding it along with a standard mixture of ^2^H-labeled amino acids, in a dual-tracer, plateau-fed test meal approach. Next, intrinsically labeled legume protein digestibility was studied with a similar dual-tracer approach, with uniformly labeled [^13^C]-spirulina as the standard, when processed differently before consumption.

**Results:**

The average digestibility of IAA in spirulina protein was 85.2%. The average IAA digestibility of intrinsically ^2^H-labeled chick pea and mung bean protein was 56.6% and 57.7%, respectively. Dehulling of mung bean before ingestion increased the average IAA digestibility by 9.9% in comparison to whole mung bean digestibility.

**Conclusions:**

An innovative, minimally invasive “dual-stable-isotope” method was developed to measure protein digestibility, in which the ingestion of an intrinsically ^2^H-labeled test protein along with a ^13^C-labeled standard protein of known digestibility allows for an accurate measure of digestion and absorption of the intrinsically labeled protein. This minimally invasive method is critical to redefining protein quality and will aid in revisiting human protein requirements in different settings and in vulnerable populations. This trial was registered at Clinical Trials Registry—India as CTRI/2017/11/010468.

## INTRODUCTION

A recent re-evaluation of human indispensable amino acid (IAA) requirements (1) showed that the daily requirement of important and potentially limiting IAAs, such as lysine, was much higher than previously thought. This has had an impact on evaluations of the quality of protein that is eaten, particularly in many low- and middle-income countries (2, 3), where cereal proteins, with a lower lysine content, form the mainstay of the daily protein intake. In turn, protein quality impacts human health, as it is important for linear growth in children (3–5).

An important and neglected dimension of protein quality is its digestibility and absorption in humans. This was hitherto measured by the orofecal balance of the fed protein (6, 7). However, the digestion and absorption of protein occurs exclusively in the small intestine, and protein synthesis by the colonic microbiome can confound orofecal nitrogen balance. Therefore, digestibility should ideally be measured via the oroileal balance (8), which is impossible to achieve without an invasive intubation or fistulation of the intestine. Oroileal digestibility also should be defined for each IAA. It has now also been proposed that protein quality should be measured for the content of each IAA, along with the digestibility of each IAA, in relation to the human requirement pattern, in what is now called the digestible indispensable amino acid score (DIAAS) (9, 10). Recently, the metabolic availability (MA) of lysine was measured in humans by a noninvasive, indicator amino acid oxidation technique (11, 12), which had been validated against animal models (13, 14). However, only one amino acid can be assessed for its MA within any experiment (11, 15).

The measurement of oroileal amino acid digestibility can be performed in a relatively minimally invasive manner, by a dual-tracer technique, in which an intrinsically isotope-labeled test protein is fed simultaneously with a different isotope-labeled “standard” protein of known digestibility. Then, the postprandial ratio of the appearance of differently labeled amino acids in the blood allows for the evaluation of the true digestion and absorption of the test protein, because the splanchnic uptake and metabolism for each amino acid can be corrected for when this ratio approach is used. This method measures amino acid appearance in the blood, but as intrinsically labeled proteins are applied, the appearance of label is reported relative to the appearance of a differently labeled isotope from a simultaneously fed standard protein. This method measures relative ileal digestibility of a test to a standard protein, which is not confounded by the digestion and absorption of amino acids from unlabeled endogenous secreted proteins. This has been called the dual-tracer method of measuring small intestinal amino acid digestibility (9) and follows the principles of other dual-tracer applications such as those used to study starch digestion (16).

The choice of a standard protein is also important, as it should be uniformly labeled and have a high digestibility. Spirulina is a cyanobacterium (*Arthrospira platensis*), available commercially as uniformly ^13^C-labeled whole cells. It is easily digestible and is considered to be a high-quality protein. Uniformly labeled [^13^C]-spirulina has been used to determine amino acid essentiality (17), and uniformly labeled [^15^N]-spirulina has also been used in a dual-tracer method, to measure the digestibility of phenylalanine (18). However, no correction was applied for tracer loss due to transamination, which is known to occur from applications of [^2^H]-phenylalanine isotopologs (19). This study aimed, first, to characterize the ileal digestibility of uniformly ^13^C-labeled spirulina protein as a standard protein in humans, in comparison with a mixture of ^2^H-labeled crystalline amino acids; and second, to demonstrate the use of this standard protein to measure the digestibility of selected legumes containing proteins that had been intrinsically labeled with ^2^H.

## METHODS

### Measurement of [^13^C]-spirulina digestibility

This study was conducted from July 2016 to March 2017 at the metabolic unit of St John's Medical College, Bangalore, India. The Institutional Ethical Review Board approved the experimental protocols. A flow chart demonstrating the screening and enrolment of participants in the study is given in **Supplemental Figure 1**. Six healthy volunteers (3 males and 3 females) aged 18–45 y, with a BMI of 18.5–25.0 kg/m^2^ were recruited, and written consent was obtained from each study participant at enrolment. Those who were pregnant, under medication, including antibiotics in the 4 wk prior to the study, or who had any food allergy, were excluded.

The test meal in which the [^13^C]-spirulina was provided consisted of rice with ghee, and a chick pea curry. The chick pea curry was pressure cooked for 25 min in a local recipe. The test meal was designed to provide a third of the day's energy, with a protein-energy ratio of ∼0.11. Uniformly ^13^C-labeled spirulina, whole cells (12 mg/kg, 97% purity; Cambridge Isotope Laboratories) and a mixture of uniformly ^2^H-labeled amino acids (1.25 mg/kg, >97% purity; Cambridge Isotope Laboratories) were mixed in the test meal homogenously. The composition of the amino acid mix is given in **Supplemental Table 1**. The amino acid mixture had a composition that was close to (but not exactly similar to) egg protein, and had, on average, a slightly higher amount of IAAs (1.06% higher). This mixture, which was commercially available, was also much more economic to use. Legume protein contributed 65% of the test meal protein, rice protein 32%, with spirulina and free amino acids, ∼3%. l-[ring-^13^C_6_]-Phenylalanine was additionally administered to measure a free amino acid absorption index, and to establish this as a measure of absorption for future experiments.

**FIGURE 1:**
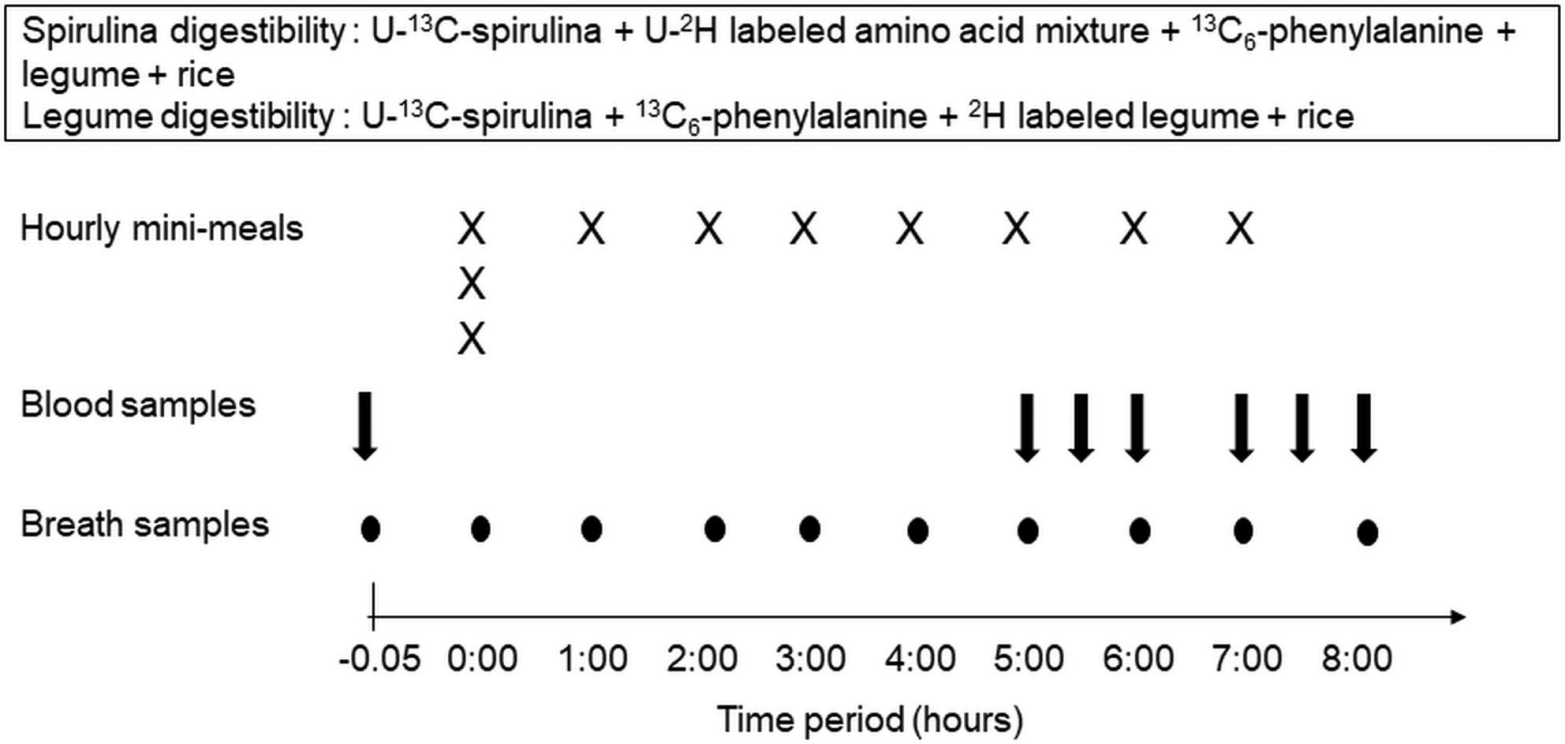
Tracer experimental protocol demonstrating the plateau feeding of test meals.

On the study day, participants reported at the metabolic unit after a 12-h fast. Body weight and height were measured by a digital beam scale (Salter) and a stadiometer (Seca GmbH). Soon after, an intravenous catheter (Jelco 22 G; Medex Medical Ltd) was inserted into the antecubital vein of one arm for the blood collection. Basal blood and breath samples were collected and feeding of the test meal was started in a plateau feeding format, where the entire meal was fed in mini-portions at constant intervals. For this, the meal was divided into 11 mini-portions, one of which was retained for analysis of isotopic enrichments. The first portion was fed as a priming dose (3 mini-portions), mixed with [^13^C]-bicarbonate (3 mg/kg, >99% puity, Cambridge Isotope Laboratories). Single mini-portions were then fed hourly for 7 h, soon after hourly collection of blood and breath samples. A detailed protocol is provided in **Figure 1**. Blood samples were collected into EDTA-coated anticoagulant tubes (Becton Dickenson) and centrifuged at 1098 g for 15 min, at 4°C immediately. The plasma was separated and stored at –80°C until analysis. Expired breath samples were collected into plain vacutainers (Becton Dickenson) and stored at room temperature until analysis. In an earlier experiment, the appropriate time points for blood collection were established by evaluating the appearance and plateau of ^2^H-labeled amino acids in the plasma, after plateau feeding of a meal containing ^2^H-labeled chick peas (see below for details on the production of labeled chick pea, methods for isotopic analyses, and digestibility result) in a test meal. This pilot experiment showed that a steady state of plasma ^2^H-labeled amino acid enrichment was achieved from 6 h after the first meal (data not shown).

Lyophilized meal samples were hydrolyzed with a 1:1, vol:vol mixture of HCl (12 M, Merck) and sodium thiosulfate (Na_2_S_2_O_3_·5H_2_O, 0.02 M, Merck), in an acid digestion vessel (Parr) for 4 h at 150°C (20). Plasma samples were ultrafiltered (30 K Amicon ultrafilter, Merck, Millipore) after spiking with nor-valine as an internal standard (30 µL of 10 mM solution, Sigma-Aldrich). Acidified samples (plasma and meal protein hydrolysates) were applied to prewashed cation exchange columns (50WX8–100 ion-exchange resin, Sigma-Aldrich), and free amino acids were eluted with a solution of ammonium hydroxide (NH_4_OH, 4 M, Merck). Eluates were dried and derivatized to their *N*-ethoxycarbonyl ethyl ester derivatives (21). Analysis of ^13^C- and ^2^H-isotopic enrichments of amino acids in the plasma and meal samples were performed by liquid chromatography-mass spectrometry (LC-MS/MS; 6495 Triple iFunnel Quadrupole, Agilent) at St John's Research Institute, Bangalore, India. The LC was equipped with a 1290 Infinity binary pump, autosampler, and a thermostatted column compartment. The LC method parameters are given in **Supplemental Table 2**. The amino acid profile of plasma and meal samples in comparison with a standard amino acid mix (AAS18, Sigma-Aldrich) were measured in a dynamic multiple reaction monitoring–based method (detailed in the **Supplemental Table 3**), and data analyses were performed with Agilent MassHunter Qualitative analysis software (version B 07.00). Breath samples were analyzed for ^13^CO_2_ enrichments by monitoring ions at *m*/*z* ratios of 44 and 45 by isotope ratio mass spectrometry (Delta V Advantage, Thermo Fisher Scientific Inc.) at St John's Research Institute, Bangalore, India. Data were processed with Isodat software (version 3.0).

Tracer:tracee ratios of carbon and hydrogen were calculated from [^13^C_n_]/[^12^C] and [^2^H_n_]/[^1^H] specific *m/z* ratios of IAAs (depending upon the maximum number of carbon and hydrogen atoms retained in the amino acid derivatives) and converted to parts per million for each amino acid. Enrichments of ^13^C and ^2^H over basal samples of IAAs in plasma and meal samples were calculated in parts per million excess (ppme). Apparent digestibility for a given amino acid for spirulina characterization was calculated from the following equation:

(1) \begin{eqnarray*} && {\rm{Apparent}}\,{\rm{digestibility}}\nonumber\\ &&\quad = \big[{\rm{plasma}}{\,^{13}}{\rm{C\! -\! IAA}}\left( {\rm {ppme}} \right)/{\rm {meal}}{\,^{13}}{\rm C} - {\rm IAA} \left( {\rm {ppme}} \right) \big]/\nonumber\\ &&\qquad \big[{\rm{plasma}}{\,^2}{\rm H }- {\rm IAA} \left( {\rm {ppme}} \right)/{\rm{meal}}{\,^2}{\rm{H - IAA}}\left( {\rm {ppme}} \right) \big] \end{eqnarray*}

The loss of the α-carbon ^2^H atom through transamination was accounted for by the summed intensities of transaminated species (M_D__–__1_) and nontransaminated species (M_D_) as observed by LC-MS/MS in the mass spectrum, by considering the maximum number of deuterium atoms (C–H bonds; N_D_) in each IAA. A transamination correction factor (F_TCF_) for each IAA was calculated to ensure equivalence of the ^2^H with the ^13^C tracer, from abundance ratios of transaminated species (M_D__–__1_) to the nontransaminated species (M_D_) at plateau, and the number of labeled deuterium atoms (N_D_) to assess the extent of transamination and to facilitate this correction in the intrinsically labeled plant protein results and in future studies that use gas chromatography-pyrolysis isotope ratio mass spectrometry (GC-P-IRMS) analysis which cannot identify individual isotopologs (see below):

(2) \begin{eqnarray*} &&{\rm{Transamination}}\,{\rm{correction}}\,{\rm{factor}}\left( {{{\rm{F}}_{{\rm{TCF}}}}} \right) = \left( {\left( {{{\rm{M}}_{{\rm{D}} - 1}}/{{\rm{M}}_{\rm{D}}}} \right) + 1} \right)\nonumber\\ &&\quad \times \left( {\left( {{{\rm{N}}_{\rm{D}}}} \right)/\left( {{{\rm{M}}_{{\rm{D}} - 1}}/{{\rm{M}}_{\rm{D}}}} \right)} \right) \times \left( {\left( {{{\rm{N}}_{{\rm{D}} - 1}}} \right) + {{\rm{N}}_{\rm{D}}}} \right) \end{eqnarray*}

### Measurement of legume protein digestibility with [^13^C]-spirulina protein as standard

A legume labeling protocol with ^2^H_2_O was conducted at the University of Agricultural Sciences, Bangalore, India, with local settings for soil, fertilizers, and water. Deuterium oxide (99.9%, Sercon Ltd) was used to intrinsically label the plant protein through strategic watering protocols during the seed development stage.

For chick peas (*Cicer arietinum*), the labeling experiment was conducted during the winter (rabi) season at a pilot scale. An Indian variety of chick pea (desi), JG11, which is consumed normally in south India, was used for this experiment. Ten pots each containing 2 healthy plants were watered gravimetrically by weighing the pots thrice daily. The containers were irrigated with normal water except on the day of ^2^H_2_O application. Approximately 2 wk after anthesis (after the plants reached 50% flowering), 400 mL of ^2^H_2_O (25%) was provided as a single watering pulse. The plants were brought to maturity and harvested. The seeds were dried and stored for further experiments.

For mung bean (*Vigna radiata*), the labeling experiment was conducted during the rainy (kharif) season. Pots were housed under an automated retractable roof, to protect plants from rain. Fifty pots, each containing 2 healthy plants, were watered gravimetrically with H_2_O. This protocol was modified from the pilot chick pea experiment to include additional pulses of ^2^H_2_O to mimic a plateau-watering protocol, i.e., each pot received a priming dose of ^2^H_2_O (25%, 400 mL) on day 0 (∼2 wk after anthesis), and thereafter, 100 mL of ^2^H_2_O (2.5%) was applied on days 2, 4, 6, and 8.

Plants were brought to maturity and harvested, and the seeds were dried and stored. Subsamples of dried chick pea and mung bean seeds were milled to fine flour in a grinder (TTK Prestige Ltd) and the protein directly hydrolysed as above. Amino acids from hydrolyzed proteins were derivatized to their *N*-ethoxycarbonyl ethyl ester derivatives (21) and their low ^2^H enrichments were analyzed by GC-P-IRMS (Delta V Advantage, Thermo Fisher Scientific Inc) at St John's Research institute, Bangalore, India. Data were acquired with the use of Isodat software (version 3.0). This amino acid derivative was chosen as it is suitable for both gas and liquid chromatography separation, so that the same processed samples could be analyzed by both mass spectrometry platforms. GC-P-IRMS measures deuterium abundance in amino acids following online high-temperature conversion of all hydrogen atoms in each derivatized molecule to H_2_ gas. Deuterium enrichment results as reported are not corrected for the number of labeled atoms, as this is unnecessary. Information about the gas chromatography method and temperature conditions are provided in **Supplemental Table 4**. Details of the GC-P-IRMS methods are provided in Supplemental Methods.

The digestibility of intrinsically labeled chick peas and mung beans was assessed by a plateau feeding experiment (as described above) that used the same recipes of ghee rice and legume curry. To evaluate the effect of dehulling on digestibility, mung beans were subjected to soaking for 7 h and heating at 70°C for 1 min, followed by manual dehulling, and then used in the same recipe to measure their digestibility. Three test meals—ghee rice with chick pea curry, ghee rice with mung bean (whole seed) curry, and ghee rice with dehulled mung bean curry—were tested on the same subject with 2-wk wash-out periods between tests. The standard protein was ^13^C-labeled spirulina, the digestibility of which was characterized with respect to a crystalline amino acid mix as described above, given at a dose of 12 mg whole cells/kg body weight. The plateau feeding protocols and sample collections were the same as in the spirulina digestibility study described above. A small quantity of l-[ring-^13^C_6_]-phenylalanine was also administered (0.05 mg/kg, 99% purity, Cambridge Isotope Laboratories) to measure a free amino acid absorption index, and to establish this as a measure of absorption for future experiments.

Legume protein digestibility was calculated as:

(3) \begin{eqnarray*} \big[ {{\rm{plasma}}{\,^2}{\rm{H\! -\! IAA}}\left( {{\rm{ppme}}} \right)/{\rm{meal}}{\,^2}{\rm{H \!-\! IAA}}\left( {{\rm{ppme}}} \right)} \big]\nonumber\\ / \big[{\rm{plasma}}{\,^{13}}{\rm{C \!-\! IAA}}\left( {{\rm{ppme}}} \right)/{\rm{meal}}{\,^{13}}{\rm{C\! -\! IAA}}\left( {{\rm{ppme}}} \right) \big] \nonumber\\ \times 100 \times {\rm{Di}}{{\rm{g}}_{{\rm{Std}}}} \times {{\rm{F}}_{{\rm{TCF}}}} \end{eqnarray*}

where Dig_Std_ is the digestibility of each IAA from the ^13^C-labeled spirulina protein and F_TCF_ is an IAA-specific term used to correct for loss of a ^2^H atom during transamination (as described above).

In addition, an absorption index was also calculated, based on the free labeled amino acid (l-[ring-^13^C_6_]-phenylalanine) added to the labeled legume and spirulina meal:

(4) \begin{eqnarray*} {\rm{Absorption}}\,{\rm{index}} &=&100 \times {\rm{plasma}}{\,^{13}}{{\rm{C}}_6} \nonumber\\ &&-\! {\rm{phe}}\,{\rm{enrichment}}\,{\rm{at}}\,{\rm{plateau}}/ \nonumber\\ &&{^{13}}{{\rm{C}}_6}-\! {\rm{phe}}\,{\rm{enrichment}}\,{\rm{in}}\,{\rm{test}}\,{\rm{meal}} \end{eqnarray*}

where ^13^C_6_-phe is the appearance of l-[ring-^13^C_6_]-phenylalanine in blood at plateau and its enrichment in the test meal.

Continuous data are presented as means ± SDs. Digestibility data were compared between groups by ANOVA, and *P* < 0.05 was considered to be statistically significant. Analyses were performed with Statistical Package for Social Sciences version 18 (SPSS Statistics for Windows, IBM).

## RESULTS

### Spirulina digestibility

**FIGURE 2:**
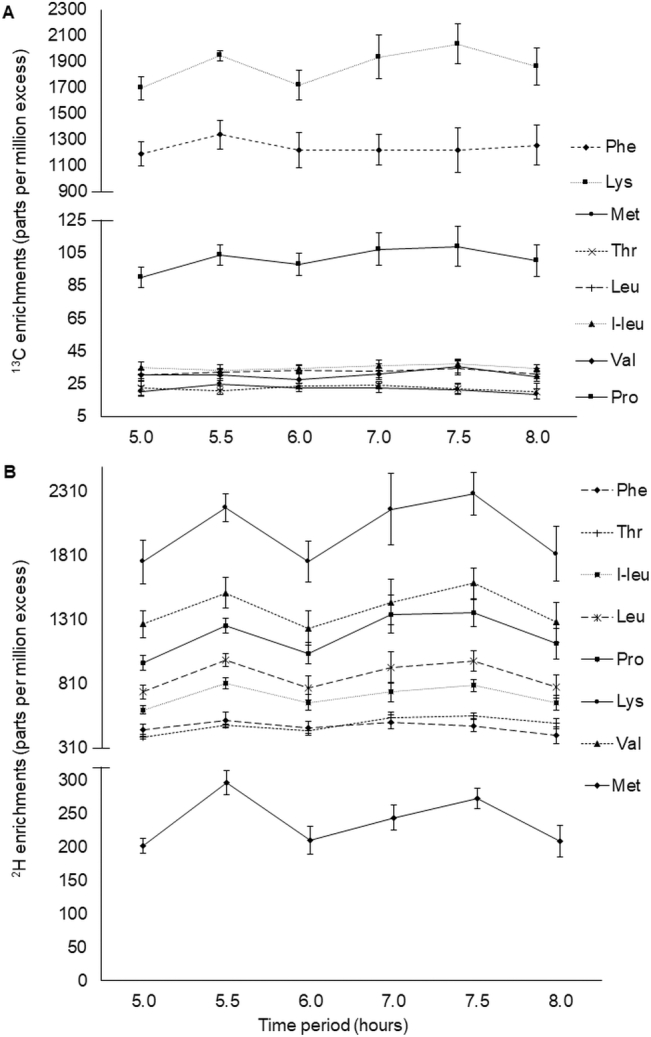
Plasma appearance of ^13^C (A) and ^2^H isotopic (B) enrichments of amino acids in parts per million excess (ppme) at plateau state. Plots represent means ± SDs of ^13^C and ^2^H enrichments appearance in plasma. I-leu, isoleucine.

Anthropometric characteristics, hemoglobin concentrations, and dietary information of the subjects (*n* = 6) are given in **Table 1**. The appearance of plasma ^13^C and ^2^H amino acid enrichments in ppme are presented in **Figure 2**A and B, and show that these reached a plateau between 5 and 8 h. The average enrichments of ^13^C- and ^2^H-enriched amino acids in the ingested meal (ppme over unlabeled meals) were: methionine, 513 ± 117, 4836 ± 572; phenylalanine 10,367 ± 1209, 3779 ± 479; threonine 328 ± 59, 5850 ± 562; lysine 17,578 ± 1806, 14,415 ± 2175; leucine 506 ± 57, 11,331 ± 868; iso-leucine 455 ± 43, 7574 ± 830; valine 336 ± 41, 13,285 ± 1279 and proline 612 ± 68, 2937 ± 331 respectively.

**TABLE 1:** Baseline characteristics of the study subjects^1^

Variables	Subjects
Age, y	19.50 ± 3.2
Education (university and above)	6 (100)
Weight, kg	55.01 ± 3.6
Height, m	1.59 ± 0.1
BMI, kg/m^2^	21.66 ± 1.3
Hemoglobin, g/dL	14.28 ± 2.6
Dietary information	
Energy, kcal/d	1565.70 ± 177
Protein, g/d	54.71 ± 13.6
Fat, g/d	51.79 ± 17.2
Carbohydrate, g/d	220.69 ± 14.9

^1^Values are means ± SDs, *n* = 6, or number (percentage).

The transamination correction factor (TCF) for each amino acid is presented in **Table 2**. This indicates that no obvious transamination was observed in lysine or threonine, which is in accord with the lack of their transaminases in humans (22). Other amino acids, such as methionine, phenylalanine, proline, and branched-chain amino acids (BCAAs; leucine, isoleucine and valine), showed transamination loss of ^2^H, with the BCAAs being highest. The order of F_TCF_ for the BCAAs was in line with their affinity for the BCAA transaminase (23).

**TABLE 2:** Transamination correction factors for loss of deuterium from indispensable amino acids^1^

Amino acid	Transamination correction factor
Methionine	1.058 ± 0.005
Phenylalanine	1.053 ± 0.006
Threonine	1.016 ± 0.002
Lysine	1.002 ± 0.002
Leucine	1.081 ± 0.002
Iso-leucine	1.070 ± 0.004
Valine	1.048 ± 0.003
Proline	1.013 ± 0.005

^1^Values are means ± SDs, *n* = 6.

The digestibility of uniformly labeled [^13^C]-spirulina protein measured by the dual-tracer technique is represented in **Table 3**. The mean digestibility of the IAAs (excluding tryptophan) was 85.2% (range 77–95%) with the highest digestibility for phenylalanine and the lowest for lysine. The digestibility varied by ∼6% between IAAs, and none of the IAA digestibilities were significantly different from each other (ANOVA, *P* = 0.74). The digestibility of the dispensable amino acid proline was relatively low, at 41.4%.

**TABLE 3:** Digestibility of amino acids in spirulina whole-cell protein^1^

Amino acid	Digestibility, %
Methionine	84.1 ± 7.6
Phenylalanine	95.3 ± 4.1
Threonine	82.5 ± 2.6
Lysine	77.5 ± 9.5
Leucine	86.0 ± 3.1
Iso-leucine	84.2 ± 2.8
Valine	87.1 ± 5.0
Proline	41.4 ± 5.7

^1^Values are means ± SDs, *n* = 6.

Oxidation of uniformly labeled [^13^C]-spirulina whole cells resulted in the ^13^C enrichment of breath CO_2_. The appearance of enrichment of ^13^CO_2_ in the breath is shown in **Supplemental Figure 2**. This showed a steady increase in enrichment over the 8 h of the plateau feeding, which was likely due to underpriming the pool with [^13^C]-bicarbonate. A 33% larger bicarbonate prime is recommended in future studies.

### Legume digestibility

Pilot experiments were conducted to establish the best time for watering the legume plants with ^2^H_2_O, by watering at different time points after 50% flowering (0, 3, 5, 7 and 15 d). The maximum ^2^H enrichments were seen at days 0 and 15 after anthesis (data not shown), and the time chosen for watering with ^2^H_2_O was 15 d after anthesis for subsequent legume labeling experiments. The average ^2^H enrichment of the chick pea IAAs, corrected for the number of labeled C atoms was 767 ppme, whereas the average corrected ^2^H enrichment of mung bean IAAs was 1204 ppme (**Supplemental Figure 3**), which was higher than that for chick peas, since additional small doses of ^2^H_2_O were provided for the mung beans.

The digestibility of intrinsically labeled chick pea, whole mung bean, and dehulled mung bean protein in a single subject, as assessed by the dual-isotopic method with [^13^C]-spirulina as the standard protein, is provided in **Table 4**. The mean IAA digestibility was 56.6% for chick pea and 57.7% for mung bean protein. However, dehulling increased the mean IAA digestibility by 9.9% compared with the mean whole mung bean digestibility. The test meals also contained l-[ring-^13^C_6_]-phenylalanine and the average enrichment of l-[ring-^13^C_6_]-phenylalanine in the meals was 2161 ± 317. A free amino acid absorption index was calculated based on the appearance of l-[ring-^13^C_6_]-phenylalanine in the plasma at plateau over the meal, and a value of 11.38 ± 2.6% obtained.

**TABLE 4:** Digestibility of amino acids in legume protein^1^

Amino acid	CP (%)	WMB (%)	DHMB (%)
Methionine	59.8	60.6	66.0
Phenylalanine	60.5	65.2	69.4
Threonine	53.8	43.6	47.2
Lysine	44.4	56.5	56.7
Leucine	68.5	62.3	72.3
Iso-leucine	68.8	76.0	80.3
Valine	64.1	64.5	78.7
Proline	33.2	32.6	36.7
Mean	56.6	57.7	63.4

^1^ *n* = 1. CP, chick pea; DHMB, dehulled mung bean; WMB, whole mung bean.

## DISCUSSION

We have demonstrated that it is possible to measure IAA digestibility through the use of an innovative dual-tracer method that uses intrinsically labeled proteins. Up to now, the measurement of protein digestibility has proved difficult, since, in addition to the invasive methods required for measurement of the oroileal balance of ingested IAAs, it was also important to exclude the contribution of endogenously secreted proteins in the intestine. Therefore, both fistulated animal studies, as well as intubation or ileostomy studies in humans, are required to correct the measured oroileal balance of protein or IAAs for the endogenous secretions, to obtain the true protein digestibility. A major advance is the use of a minimally invasive dual-tracer method, where the digestibility of an intrinsically labeled test protein is measured against a differently labeled standard protein, by evaluating the ratios of test to standard IAA enrichments in the food and their appearance in blood. As test and standard proteins are delivered simultaneously, it is assumed that their splanchnic extraction terms will be the same. In addition, since this method only measures the appearance of labeled amino acids from the intrinsically labeled test and standard proteins, it is not confounded by endogenous protein secretion, and is hence a measure of true ileal digestibility. The same principle has been used in the context of measuring starch digestion in vivo (16). The dual-tracer approach had been used in earlier studies of protein digestion, albeit for a single amino acid, where phenylalanine digestibility was measured by the dual-tracer method in humans with cystic fibrosis, with the use of uniformly labeled [^15^N]-spirulina (18), although no correction was applied in this study to account for tracer loss by transamination. The use of a single tracer has also enabled the ileal digestibility of intrinsically ^15^N-labeled pea protein to be measured by intubation (24). Tracer-based approaches have also been used to study the digestibility of ^13^C- and ^15^N-labeled egg protein (25), where the ileal effluent was collected in ileostomates and analyzed for their residual labeled protein content, in a classical oroileal balance. Other intrinsically labeled proteins, such as milk, soy, and pea protein, have also been used in human intestinal intubation and perfusion methods to measure their digestibility, but this method is highly invasive, and has thus far yielded the digestibility of specific, and not all, IAAs (24, 26, 27), and falls short of informing the DIAAS protein quality index. An indicator amino acid oxidation method has also been proposed to measure the metabolic availability (including digestion, absorption, and utilization) of specific IAAs. Although noninvasive, since it is a breath test, a disadvantage is that subjects need to be tested repeatedly, and the MA of only one IAA can be studied within an experiment (11, 12, 15).

In the present study, the following 3 advances were made: *1*) intrinsically labeled test protein, obtained by watering plants (legumes) with a ^2^H_2_O pulse protocol during their flowering, such that all the IAAs in their seeds were uniformly labeled to an extent that was practical for human testing by the dual-tracer method, was used; *2*) the relative ileal digestibility of all the IAAs (except tryptophan and histidine; tryptophan information could be recovered by processing a test meal aliquot by unconventional hydrolysis) was measured by the minimally invasive dual-tracer method, allowing these values to be used in a DIAAS framework, which corrects for each IAA digestibility when assessing protein quality; *3*) the relative ileal IAA digestibility of a relatively high-quality spirulina protein was characterized for future use as a standard protein in such experiments. This is useful as a secure supply of uniformly labeled [^13^C]-spirulina is available commercially. The findings of the present study indicate that intrinsic labeling of legumes with ^2^H_2_O produced sufficient enrichment for human experimental protocols to be conducted.

The average IAA digestibility of a relatively high-quality standard uniformly ^13^C-labeled spirulina protein was 85.2%. The phenylalanine digestibility was 95%, which can be compared to 80% reported earlier for phenylalanine (18) in a similar dual-tracer human study. However, the latter would likely have underestimated phenylalanine digestibility as tracer loss through transamination was not taken into account. Average IAA digestibility was similar to that obtained in a study in growing rabbits (28), and was in the range of other reports (29, 30). Specific IAA digestibilities did not vary significantly from each other, and the interindividual variability in the healthy men studied varied from 3.2% to 12.3%. When intrinsically ^2^H-labeled legumes (chick pea and mung bean) were prepared according to a local recipe, their average IAA digestibility was similar (60.6%); lysine had the lowest digestibility (44.4%) for chick pea, whereas threonine had the lowest digestibility for whole (43.6%) or dehulled mung bean (47.2%). Dehulling the mung bean before preparation increased its average IAA digestibility by 9.9%. An increase was expected since food processing can improve digestibility (31) and dehulling also decreases antinutritional factors, such as antitrypsins, phytic acid, and tannins, which are concentrated in the seed hull (32). Furthermore, this also highlights the ability of the method to measure relatively small differences in digestibility and to compare different plant protein sources as well as different processing and preparation techniques.

The advantage of the dual-tracer protocol over other methods is that the plasma appearance of labeled IAAs from the test and reference or standard protein in relation to their enrichment in the ingested meal gives a unique measure of the ileal IAA digestibility. To reduce the number of blood samples, a primed plateau-feeding protocol was established after pilot testing, and it was evident that the time taken to reach such a plateau was a minimum of 6 h in adults, such that the duration of the experiment was 8 h. Another factor to be considered is first-pass splanchnic metabolism, where a proportion of absorbed amino acids are lost to protein synthesis and catabolism, whereas protein breakdown introduces unlabeled IAAs. Also, transamination is an equilibrium process which is especially active in the liver, causing loss of one ^2^H atom, such that the digestibility assessment requires a transamination correction. The transamination rate was significant but ^2^H enrichment suffers a modest change as transamination acts only on the H atom at carbon atom 2 position. Use of highly ^2^H-substituted tracers with LC-MS/MS analysis informed this correction term. In contrast, the sensitive GC-P-IRMS technique does not give positional information, but is useful in determining accurate legume-derived amino acid appearance values once a transamination correction factor has been generated. A correction for transamination is unnecessary when ^13^C tracers are used, but a large correction term would be required for most IAAs if ^15^N were used. Over the experimental period of 8 h, most IAAs underwent transamination to some degree, with the BCAAs having the highest F_TCF_. As only 1 of 10 carbon-bound H atoms in leucine is subject to transamination, this factor has a maximum value of 1.11. Our results with respect to BCAAs transamination affinity are in line with expectations from enzyme kinetics (23). Transamination also precludes the use of ^15^N-labeled proteins as a general IAA tracer in the dual-tracer method, particularly for BCAAs, which are known to undergo rapid and reversible transamination (33, 34).

Protein quality evaluation has previously been based on protein digestibility, measured through orofecal N balances, and the amino acid score, or the IAA content of the protein in relation to the age-specific requirement pattern. This was called the protein digestibility-corrected amino acid score (PDCAAS), and was central to assessing protein and IAA requirements. However, the PDCAAS approach does not take the individual amino acid digestibility into consideration (35), and hence, the new DIAAS system has been recommended for protein quality evaluation, since it is defined for each individual IAA, at the level of the ileum. However, for the DIAAS to be accepted for global use, particularly in public health nutrition, human digestibility measurements, at different ages and vulnerabilities, and in different environments, are required (8). Moreover, the DIAAS method more accurately describes the value of protein ingredients in a meal, because it does not truncate scores to a maximum of 1.0, as the PDCAAS does. Since the novel dual-tracer method provides data on relative ileal digestibility at the level of individual IAAs, in a minimally invasive way and in human subjects, it satisfies the requirements for the introduction of DIAAS, and should prove useful in informing protein quality in a number of key plant protein sources. It is somewhat expensive to implement at present, but is intended for use in key groups on representative diets that will inform protein demand in populations. The limitations of the study include the loss of tryptophan during acid hydrolysis, which can be addressed by modified processing of the test meals. In addition, measurement of legume digestibility by the dual-tracer method should be extended to sufficient human volunteers to evaluate its variability. Finally, this method has not yet been tested in animal validation studies (this is in planning), in contrast to the MA method, which was validated against reference methods in animals (13, 14).

One key factor in the measurement of digestibility for global evaluations is the presence of environmental enteric dysfunction, which can occur in those living in poor and challenging environments. In this pathophysiologic state, where persistent immune activation and increased intestinal permeability is widespread (36), it is thought that there may also be reduction in the ability to digest and absorb protein, thereby impacting, for example, linear growth. It has been shown that IAA requirements are increased in disadvantaged populations living in poor environments (37–39); part of this may be due to poor digestion and absorption, since interventions such as deworming reduced the IAA requirement towards the normal requirement in healthy subjects in better environments. The dual-tracer technique allows for measurements of protein digestibility in such populations, and may thus aid in revisiting protein requirements in settings where plant- and legume-based diets are usually consumed due to vegetarianism, or simply due to limited resources.

In conclusion, we have demonstrated the use of a minimally invasive dual-tracer method of measuring protein digestibility, which is consistent with all the criteria required for the DIAAS protein quality index. We have also defined the digestibility of a standard protein for future tests. Furthermore, as this method exploits mass spectrometry for amino acid analysis, it measures unmodified amino acids, and is not compromised by amino acid modifications that may have occurred during processing and preparation (10).

## Supplementary Material

Supplementary Data

## Notes

Supported by the Wellcome Trust/DBT India Alliance in the form of a Margdarshi fellowship to AVK.

Supplemental Methods, Supplemental Tables 1–4, and Supplemental Figures 1–3 are available from the “Supplementary data” link in the online posting of the article and from the same link in the online table of contents at https://academic.oup.com/ajcn/.

Abbreviations used:

BCAA: branched-chain amino acid
DIAAS: digestible indispensable amino acid score
GC-P-IRMS: gas chromatography-pyrolysis isotope ratio mass spectrometry
IAA: indispensable amino acid
MA: metabolic availability
PDCAAS: protein digestibility-corrected amino acid score
ppme: parts per million excess
F_TCF_: transamination correction factor
