# Supplementary material for: Measurement of protein digestibility in humans by a dual-tracer method
Source: Am J Clin Nutr. 2018 May 15;107(6):984–91. doi: 10.1093/ajcn/nqy062 (PMC6179135; doi:10.1093/ajcn/nqy062)
Supplement: Supplementary Data [file nqy062_supp.zip › ajcn164970-file001.docx]

**Online supporting material**

**Measurement of protein digestibility in humans by a dual tracer method**

Sarita Devi, Aneesia Varkey, MS Sheshshayee, Thomas Preston, Anura V Kurpad

The composition of U-^2^H labeled amino acid mixture used for spirulina digestibility experiments is given below in **Supplemental Table 1.**

**Detailed LC-MS/MS Assay Conditions**

1. **Agilent 1290 Infinity HPLC**

Autosampler

- - Temperature: 4°C
  - Injection Volume: 1 μL
  - Needle Wash Solution: Methanol

Column

- - ZORBAX Eclipse Plus Phenyl Hexyl C18 column, 2.1 x 150 mm ID, 1.8 μm particle size, Agilent Technologies
  - Temperature: 45°C
  - Maximum Pressure: 1200 bar

Binary Pump

- - Flow Rate: 0.4 mL/min
  - Solvent A: 0.1% Formic acid with 10 mM ammonium formate in H_2_O
  - Solvent B: 80:20, Methanol: IPA with 0.1% Formic acid and 10 mM ammonium formate

Details of LC gradients are given below in **Supplemental** **Table 2.**

1. **Agilent 6495 Triple iFunnel Quadrupole Mass Spectrometer**
   - Gas Temperature: 250˚C
   - Gas Flow: 11 L/min.
   - Nebulizer: 30 psi
   - Sheath Gas Temperature: 400˚C
   - Sheath Gas Flow: 11 L/min.
   - Capillary Voltage: 3500 V
   - Nozzle Voltage: 0 V
   - Electrospray ionization: Positive

Details of dynamic MRM transitions are given below in **Supplemental** **Table 3.**

1. **Gas Chromatography-Pyrolysis-Isotope Ratio Mass Spectrometry**

**Assay Conditions**

Autosampler

- - Injector Temperature: 280^°^C
  - Inlet mode: splitless mode
  - Injection Volume: 5 μL
  - Needle Wash Solution: Isooctane

Column

- - DB-17HT, 60 m, 0.32 mm inner diameter, 0.15 µm film thickness, J&W Scientific, CA, USA
  - Carrier gas: Helium
  - Gas flow: 0.8 mL/min

GC-IsoLink/ConFlow IV interface

- - HTC reactor temperature: 1400°C
  - Backflush helium flow: 1.5 bar
  - Reference gas (Hydrogen): 0.8 bar
  - Helium flow: 1.0 bar

GC temperature programme conditions are given below in **Supplemental** **Table 4.**

**Supplementary Table 1.** Composition of ^2^H labeled amino acid mixture

| Amino acids | Purity, % | Percentage in mix |
| --- | --- | --- |
| L-Lysine:2HCl | 98 | 12 |
| L-Leucine | 98 | 9 |
| L-Glutamic Acid | 97-98 | 9 |
| L-Aspartic Acid | 98 | 8 |
| L-Alanine | 98 | 6 |
| L-Arginine:HCl | 98 | 6 |
| L-Glutamine | 97 | 5 |
| Glycine | 98 | 5 |
| L-Asparagine:H_2_O | 94 | 5 |
| L-Proline | 97-98 | 5 |
| L-Phenylalanine | 98 | 4 |
| L-Valine | 98 | 4 |
| L-Serine | 98 | 4 |
| L-Threonine | 98 | 4 |
| L-Tyrosine | 98 | 3 |
| L-Isoleucine | 98 | 3 |
| L-Tryptophan | 97-98 | 3 |
| L-Cysteine | 98 | 3 |
| L-Histidine:HCl:H_2_O | 98 | 1 |
| L-Methionine | 98 | 1 |

Source: "cell free" amino acid mix (20 AA) (U-D, 98%) DLM-6819 (Adapted from Cambridge Isotope Laboratories, USA)

**Supplemental** **Table 2.** LC gradient profile with retention time

| **Segment** | **Time (min)** |  | **% B** | **Flow Rate (mL/min)** |
| --- | --- | --- | --- | --- |
| 0 (Start) | 0.0 |  | 5 | 0.4 |
| 1 | 3.0 |  | 25 | 0.4 |
| 2 | 25.0 |  | 60 | 0.4 |
| 3 | 30.0 |  | 95 | 0.4 |
| 4 | 34.0 |  | 100 | 0.4 |
| 5 | 38.0 |  | 75 | 0.4 |
| 6 | 40.0 |  | 50 | 0.4 |
| Re-equil. | 43.0 |  | 5 | 0.4 |

**Supplemental** **Table 3.** Dynamic MRM transitions

| **Compound Name** | **Precursor Ion** | **Product Ion** | **MS2 Res** | **Ret Time (min)** | **Fragmentor** | **Collision Energy** | **Cell Accelerator Voltage** |
| --- | --- | --- | --- | --- | --- | --- | --- |
| I-Leu | 232.15 | 158.12 | Unit | 16.2 | 380 | 5 | 7 |
| I-Leu ^13^C_5_ | 237.15 | 163.12 | Unit | 16.2 | 380 | 5 | 7 |
| I-Leu ^2^H_10_ | 242.15 | 168.12 | Unit | 16.2 | 380 | 5 | 7 |
| I-Leu ^2^H_9_ | 241.15 | 167.12 | Unit | 16.2 | 380 | 5 | 7 |
| Leu | 232.15 | 158.12 | Unit | 16.8 | 380 | 5 | 7 |
| Leu ^13^C_5_ | 237.15 | 163.12 | Unit | 16.8 | 380 | 5 | 7 |
| Leu ^2^H_10_ | 242.15 | 168.12 | Unit | 16.8 | 380 | 5 | 7 |
| Leu ^2^H_9_ | 241.15 | 167.12 | Unit | 16.8 | 380 | 5 | 7 |
| Lys | 319.19 | 273.2 | Unit | 13.8 | 380 | 7 | 7 |
| Lys ^13^C_6_ | 325.19 | 279.2 | Unit | 13.8 | 380 | 7 | 7 |
| Lys ^2^H_8_ | 327.19 | 281.2 | Unit | 13.8 | 380 | 7 | 7 |
| Lys ^2^H_9_ | 328.19 | 282.2 | Unit | 13.8 | 380 | 7 | 7 |
| Met | 250.11 | 176.07 | Unit | 12.5 | 380 | 5 | 7 |
| Met ^2^H_7_ | 257.11 | 183.07 | Unit | 12.5 | 380 | 5 | 7 |
| Met ^13^C_4_ | 254.11 | 180.07 | Unit | 12.5 | 380 | 5 | 7 |
| Met ^2^H_8_ | 258.11 | 184.07 | Unit | 12.5 | 380 | 5 | 7 |
| Phe | 266.14 | 220.2 | Unit | 18.7 | 380 | 3 | 7 |
| Phe ^13^C_9_ | 275.14 | 229.2 | Unit | 18.7 | 380 | 3 | 7 |
| Phe ^13^C_6_ | 272.14 | 226.2 | Unit | 18.7 | 380 | 3 | 7 |
| Phe ^2^H_7_ | 273.14 | 227.2 | Unit | 18.7 | 380 | 3 | 7 |
| Phe ^2^H_8_ | 274.14 | 228.2 | Unit | 18.7 | 380 | 3 | 7 |
| Pro | 216.12 | 142.09 | Unit | 11.2 | 380 | 5 | 7 |
| Pro ^13^C_4_ | 220.12 | 146.09 | Unit | 11.2 | 380 | 5 | 7 |
| Pro ^2^H_6_ | 222.12 | 148.09 | Unit | 11.2 | 380 | 5 | 7 |
| Pro ^2^H_7_ | 223.12 | 149.09 | Unit | 11.2 | 380 | 5 | 7 |
| Thr | 220.12 | 146.1 | Unit | 5.89 | 380 | 5 | 7 |
| Thr ^13^C_3_ | 223.12 | 149.1 | Unit | 5.89 | 380 | 5 | 7 |
| Thr ^2^H_4_ | 224.12 | 150.1 | Unit | 5.89 | 380 | 5 | 7 |
| Thr ^2^H_5_ | 225.12 | 151.1 | Unit | 5.89 | 380 | 5 | 7 |
| Val | 218.14 | 144.1 | Unit | 12.6 | 380 | 5 | 7 |
| Val ^13^C_4_ | 222.14 | 148.1 | Unit | 12.6 | 380 | 5 | 7 |
| Val ^2^H_7_ | 225.14 | 151.1 | Unit | 12.6 | 380 | 5 | 7 |
| Val ^2^H_8_ | 226.14 | 152.1 | Unit | 12.6 | 380 | 5 | 7 |

**Supplemental** **Table 4.** GC temperature programme conditions

| **Segment** | **Rate (^°^C/min)** | **Temperature (^°^C)** | **Hold time**  **(min)** |
| --- | --- | --- | --- |
| Initial |  | 80.0 | 2.0 |
| 1 | 25.0 | 150.0 | 0.0 |
| 2 | 2.5 | 200.0 | 0.0 |
| 3 | 1.0 | 220.0 | 0.0 |
| 4 | 4.0 | 300.0 | 0.0 |
| 5 | 20.0 | 335.0 | 7.0 |

All subjects completed the study and data collected from (n= 6) subjects

Assessed for eligibility (n=8)

Excluded (n=2)

- Not meeting inclusion criteria (n=2)

Enrolled (n=6)

Allocated to tracer experimental protocol, n=6, males and females (in 1:1)

Dual stable –isotope study (n=6)

**Supplemental Figure 1.** CONSORT Flow chart

**
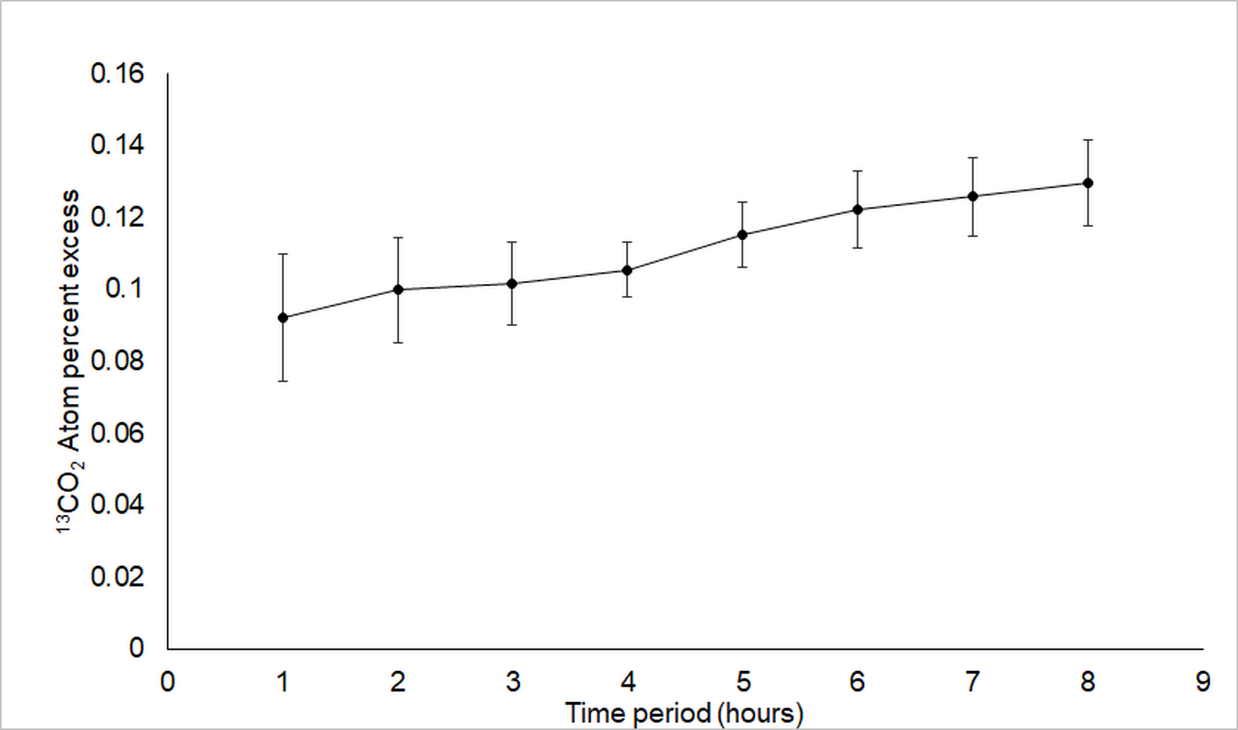
**

**Supplemental Figure 2.** Appearance of ^13^CO_2_ enrichments in breath from n=6 subjects studied for spirulina digestibility. Graph represents mean ± SD of atom percent excess (APE).

**
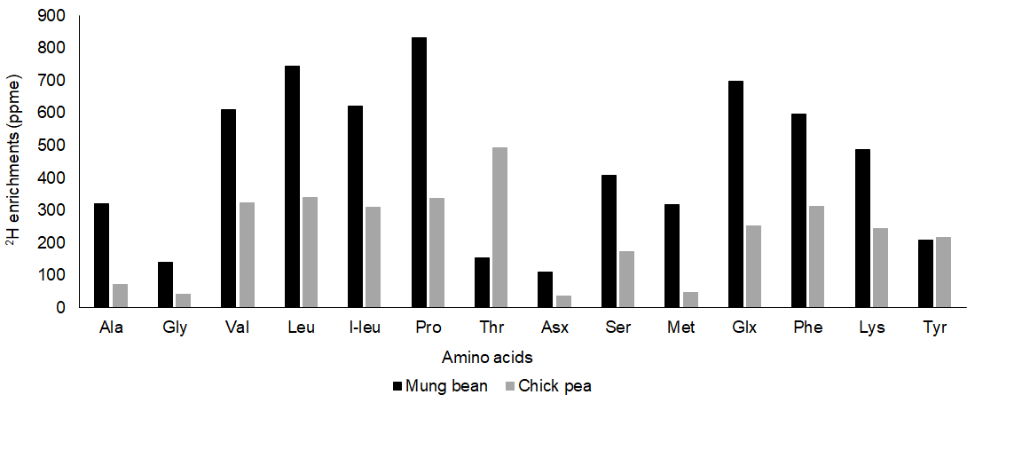
**

**Supplemental Figure 3.** Frequency plot showing average ^2^H enrichments of amino acids in pilot mung bean and chick pea

Ala: Alanine, Gly: Glycine, Val: Valine, Leu: Leucine, I-leu: Iso-leucine, Pro: Proline, Thr: Threonine, Asx: Aspartic acid + Asparagine, Ser: Serine, Met: Methionine, Glx: Glutamic acid + Glutamine, Phe: Phenylalanine, Lys: Lysine, Tyr: Tyrosine
