# Supplementary material for: Measurement of protein digestibility in humans by a dual-tracer method
Source: Am J Clin Nutr. 2018 May 15;107(6):984–91. doi: 10.1093/ajcn/nqy062 (PMC6179135; doi:10.1093/ajcn/nqy062)
Supplement: Supplementary Data [file nqy062_supp.zip › ajcn164970-file003.doc]

**CONSORT Flow chart**

Assessed for eligibility (n=8)

Excluded (n=2)

- Not meeting inclusion criteria (n=2)

Dual stable –isotope study (n=6)

Allocated to tracer experimental protocol, n=6, males and females (in 1:1)

Enrolled (n=6)

All subjects completed the study and data collected from (n= 6) subjects
